# Design of a Plant-Based Yogurt-Like Product Fortified with Hemp Flour: Formulation and Characterization

**DOI:** 10.3390/foods12030485

**Published:** 2023-01-20

**Authors:** Marco Montemurro, Michela Verni, Carlo Giuseppe Rizzello, Erica Pontonio

**Affiliations:** 1 Department of Soil, Plant and Food Science, University of Bari Aldo Moro, 70126 Bari, Italy; 2 National Research Council of Italy, Institute of Sciences of Food Production (CNR-ISPA), 70126 Bari, Italy; 3 Department of Environmental Biology, “Sapienza” University of Rome, 00185 Rome, Italy

**Keywords:** yogurt-like, hemp, lactic acid bacteria, fermentation, nutritional characterization, shelf-life

## Abstract

Plant-based milk alternatives have gained massive popularity among consumers because of their sustainable production compared to bovine milk and because of meeting the nutritional requests of consumers affected by cow milk allergies and lactose intolerance. In this work, hemp flour, in a blend with rice flour, was used to design a novel lactose- and gluten-free yogurt-like (YL) product with suitable nutritional, functional, and sensory features. The growth and the acidification of three different lactic acid bacteria strains were monitored to better set up the biotechnological protocol for making the YL product. Hemp flour conferred the high fiber (circa 2.6 g/100 g), protein (circa 4 g/100 g), and mineral contents of the YL product, while fermentation by selected lactic acid bacteria increased the antioxidant properties (+8%) and the soluble fiber (+0.3 g/100 g), decreasing the predicted glycemic index (−10%). As demonstrated by the sensory analysis, the biotechnological process decreased the earthy flavor (typical of raw hemp flour) and increased the acidic and creamy sensory perceptions. Supplementation with natural clean-label vanilla powder and agave syrup was proposed to further decrease the astringent and bitter flavors. The evaluation of the starter survival and biochemical properties of the product under refrigerated conditions suggests an estimated shelf-life of 30 days. This work demonstrated that hemp flour might be used as a nutritional improver, while fermentation with a selected starter represents a sustainable and effective option for exploiting its potential.

## 1. Introduction

The demand for plant-based meat and dairy alternatives as potential substitutes for animal-sourced foods and pathways to transition to more sustainable diets is continuously growing [1]. Due to the sustainability issues and health concerns related to animal fat intake, as well as lactose intolerance and allergies to milk proteins, plant-based alternatives to dairy milk are gaining a market share relative to cow’s milk in the United States, Europe, Australia, and New Zealand, and are forecasted to grow at a rate of 8–15% [2]. Besides the sustainability issue, lactose intolerance affects about 57% of the worldwide population (mainly in Africa and Asia) [3]. Similarly, milk protein allergy is the most prevalent food allergy in children, with symptoms ranging from mild to severe (e.g., digestive, dermatological, or respiratory) and a prevalence of slightly lower than 5% [4]. Moreover, milk proteins are also considered less sustainable compared to plant-based ones from an ecological, economical, and social point of view [5]. Indeed, the increase in alternative plant-based products can reduce malnutrition and can contribute to improving food production sustainability and a reduced environmental impact [5,6]. However, consumers still need to be informed about the characteristics of plant-based alternatives, while industries should find new products to meet the acceptability of consumers. Thus, research on fermented plant-based products as an alternative to conventional yogurt is expanding [7,8].

Plant-based yogurt-like products (PBYLPs) are vegetable products with comparable texture and sensory attributes to regular yogurt, as well as the capacity to harbor live lactic acid bacteria (LAB) for long-term preservation [8]. Many PBYLPs are produced by fermenting aqueous extracts or flour-water suspensions of cereal, pseudocereals, legumes, nut flours, or fruit pulps [9]. In recent years, several efforts to obtain a PBYLP protein structure similar to that of yogurt have been made. However, several issues related to product texture and structure still need to be solved because of the low content of proteins and the variable coagulation characteristics. Moreover, due to the acidification during fermentation processes, the instability of the plant protein structure might result in a weakening of the product structure and a separation of the aqueous phase during storage [10], thus requiring the addition of structuring agents and emulsifiers [11].

Cereals are an excellent source of vitamins, minerals, and fiber [12,13], but the rising frequency of celiac sprue and other disorders connected to gluten consumption has led to the research of gluten-free flour alternatives [14]. Nowadays, PBYLPs made from soy or coconut represent the main commercially available products in the market, and different alternatives are requested by consumers [15]. In this scenario, the growing consumer interest in the health benefits of food is driving the food industry towards the use of alternative protein sources, such as hemp [16]. Recently, the nutritional and functional potential of hemp has been examined extensively [17], reporting lipids with a unique and perfectly balanced fatty acids composition, proteins that are easy to digest and are rich in essential amino acids, and carbohydrates that are mainly represented by dietary fiber (mainly insoluble), as well as an abundance of vitamins and minerals [17]. Hemp seeds are mainly characterized by 25–35% oil, 20–25% protein, 20–30% carbohydrates, and 10–15% insoluble fiber, vitamins, and minerals [18]; however, in addition to the nutritional value, they are also rich in natural antioxidants and other bioactive components, such as bioactive peptides, phenolic compounds, tocopherols, carotenoids, and phytosterols [18]. However, the presence of antinutritional factors (ANF) (e.g., phytic acid) and their sensory and nutritional effects continue to be the most significant challenge for industrial applications. In order to overcome this problem, the fermentation of hemp flour with selected LAB was previously recognized as one of the most effective options to promote its use in cereal-based food [19,20,21]. The use of properly selected LAB can also improve the nutritional and functional values of hemp flour, as previously reported by Pontonio et al. [22].

In this work, a novel yogurt-like product, made up of a mixture of rice and hemp flour, was designed, and the biotechnological protocol was set up. Two LAB strains, previously selected for their capability to improve the functional properties of hemp flour [22], together with an exopolysaccharides (EPS)-producing strain [23], were used to ferment the rice/hemp substrate, which was characterized before and after bioprocessing according to the main chemical, biochemical, nutritional, and functional properties. Moreover, the shelf-life of the product under 4 °C storage for 30 days and the sensory properties of the formulation (also including agave syrup and vanilla powder) were evaluated.

## 2. Materials and Methods

### 2.1. Flours and Microbial Strains

Commercial rice (Bioalimenta S.r.l., Fara San Martino, Italy) and hemp (Canapuglia Fattorie, Progetto Canapuglia (Conversano, Italy) flour were used in this study. The proximal composition (g/100 g) of the flours was as follows: rice (total carbohydrates 72.0, of which sugar: 2.0, and dietary fibers: 3.0 and lipids: 2.5, of which saturated: 0.5, proteins: 8.0, and salt: 0.01); hemp (total carbohydrates 26.2, of which sugar: 3.1 and dietary fibers: 20.7 and lipids: 8.4, of which saturated: 0.9, proteins: 28.0, and salt: 0.03). The rice flour was remilled by the laboratory mill Ika-Werke M20 (GMBH, and Co. KG, Staufen, Germany) to obtain granulometry < 150 μm. Agave syrup (total carbohydrates 78.6, of which sugar: 77.4 and dietary fibers: 1.2) (Ristora, Montichiari, Italy) and vanilla Bourbon powder (total carbohydrates: 20.0, of which sugar: 5.8, and dietary fibers: 4.5 and lipids: 12.5, of which saturated: 1.3, proteins: 8.8, and salt: 0.01) (Rapunzel, Legau, Germany) were purchased from local suppliers. *Lactiplantibacillus plantarum* 18S9 and *Leuconostoc mesenteroides* 12MM1, belonging to the culture collection of the Department of Soil, Plant and Food Sciences (University of Bari, Bari, Italy), previously isolated from hemp flour [20] and selected based on protechnological characteristics and the capability to increase their antioxidant properties [22], were used in this study. Moreover, *Leuconostoc pseudomesenteroides* DSM20193, widely characterized due to their capability to produce EPS [23], were included in the study. Strains were routinely propagated in De Man, Rogosa, and Sharpe (MRS, Oxoid, Basingstoke, Hampshire, UK) at 30 °C for 24 h. When used as starters for fermentation, LAB were cultivated until the late exponential phase of growth was reached (circa 16 h), harvested by centrifugation at 9000× *g* at 4 °C for 10 min, washed twice in 50 mM phosphate buffer (4 °C, pH 7.0), resuspended in the tap water (final cell density of circa 7 log cfu/g), and used to make the yogurt-like product.

### 2.2. Biotechnological Protocol Optimization

Different ratios between the flour and water and different hemp flour amounts (added as partially replacement of the rice) were tested to achieve a suitable viscosity for the substrate. In detail, rice flour was used at 20, 25, 28, and 30% in tap water, and hemp flour was used in substitution of the 15, 20, 22, and 25% of the rice flour (Table S1). Mixtures of flour and water were homogenized with an Oster 6805 (Jarden Consumer Solutions Ltd., Cheadle, UK) mixer (ngYL) and subjected to a gelatinization process at 80 °C for 15 min, as described by Lindeboom et al. [24]. Then, the gelatinized mixtures (gYL) were cooled at 4 °C until the temperature of 30 °C prior the inoculum of *La. plantarum* 18S9, *Le. mesenteroides* 12MM1, and *Le. pseudomesenteroides* DSM20193 (ratio 1:1:1). The initial cell density of each strain was *circa* 6.2 log10 log/mL. Fermentation was carried out at 25 °C for 16 h (YL). At the end of fermentation, YL was cooled to 4 °C in 5 min, packaged in a glass jar, and analyzed within 2 h after fermentation.

### 2.3. Process Monitoring

The best formulation selected in the previous step was 30% of mixed flour (23.4 and 6.6% of the rice and hemp flours, respectively) and 70% of drinkable tap water.

The apparent viscosity of gYL and YL was monitored using 35 mL of product, previously kept at room temperature (25 °C) for 30 min, using the Viscotech Myr VR 3000 rotational viscometer equipped with L4 probe (TQC, Capelle aan den Ijssel, Netherlands) at 100 rpm. The pH of the gYL and YL was determined with a pH-meter M.507 (Crison, Milan, Italy) equipped with a food penetration probe. Mixtures were also characterized for the presence of presumptive LAB, yeasts, molds, and enterobacteria. In detail, LAB were determined on MRS (Oxoid, Basingstoke, UK) supplemented with cycloheximide (0.1 g/L), incubating the plates at 30 °C for 48 h. Yeast and molds were plated on Sabouraud Dextrose Agar (SDA, Oxoid, Basingstoke, UK) supplemented with chloramphenicol (0.1 g/L) and potato dextrose agar (PDA, Oxoid, Basingstoke, UK), respectively, at 25 °C for 48 h. Violet red bile glucose agar (VRBGA, Oxoid, Basingstoke, UK) was used to enumerate the total enterobacteria at 37 °C for 24 h.

The total titratable acidity (TTA) was determined on 10 g of product homogenized with 90 mL of distilled water and expressed as a quantity (mL) of the 0.1 M NaOH needed to reach a pH of 8.3.

During the fermentation process, the pH and LAB cell density were monitored every 2 h for 16 h. The kinetics of the growth and acidification were modeled through a modified Gompertz equation. For the kinetic of acidification modeling, the modifications proposed by Bevilacqua et al. [25] were applied as follows: the negative value of μmax was intended as the higher acidification rate (Vmax, ΔpH/h); λ, the lag phase, as the time (h) before the start of the decrease in the pH value (α; metabolic adaptation time), and A as the maximum decrease in pH (ΔpH) [25].

### 2.4. Biochemical Characterization of Yogurt-Like Product

gYL and YL were characterized for the organic acids and the total free amino acids contents. In detail, Tris-HCl extracts (water-soluble extracts, WSE) were prepared according to Weiss et al. [26] and employed for peptides, single, total free amino acids (TFAA), and organic acids analyses. The peptide concentration was determined by the *o*-phtaldialdehyde method, as described by Church et al. [27], on the supernatants treated with trifluoroacetic acid (0.05% *wt*/*vol*) and dialyzed (cutoff 500 Da) to remove the proteins and FAA, respectively. TFAA were determined by a Biochrom 30+ series Amino Acid Analyzer (Biochrom Ltd., Cambridge Science Park, UK) with a Li-cation-exchange column (20 by 0.46 cm inner diameter). For lactic and acetic acid concentrations, the kits K-DLATE and K-ACET (Megazyme, Bray, Ireland) were used, following the manufacturer’s instructions. The fermentation quotient was calculated as the molar ratio between the lactic and acetic acids. The antioxidant properties of gYL and YL were evaluated by ABTS^+^ assay in WSE and methanolic extract (ME). The latter were obtained from suspensions obtained by 3 g of each sample in 30 mL of 80% methanol. The suspensions were purged and mixed with a stream of nitrogen for 30 min and then centrifuged at 6490 rpm for 20 min. The evaluation of the scavenging activity against the ABTS^+^ radical was evaluated by using the CSO790 Kit (Sigma-Aldrich, Darmstadt, Germany), following the manufacturer’s instructions for both the methanol and aqueous extracts. The scavenging activity was expressed as Trolox equivalents.

### 2.5. Nutritional Characterization of Yogurt-Like

gYL and YL were evaluated for their composition in macro- and micronutrients. In detail, the ash, moisture, and protein contents were determined according to ISO 2171: 2007, ISO 712: 2010, and ISO 16634: 2016 (part 2), respectively. The fat was determined according to the method described in the Italian D.M. n. 4 of 23 July 1994, while the saturated fatty acids were analyzed after determining methyl esters according to Reg. 2568/1991. Carbohydrates were determined from the difference in the nutrients according to Legislative Decree n. 77 of 16 February 1993, while total fiber was carried out according to AOAC 985.29. The sugars (fructose, glucose, galactose, lactose, maltose, and sucrose) were evaluated using the K-FRUGL, K-LACGAL, and K-MASUG (Megazyme, Bray, Ireland) kits, respectively. The contents of calcium, iron, phosphorus, magnesium, potassium, and zinc were determined according to the AOAC method 984.27. The vitamins were evaluated by the Food Safety Lab (Corato, Italy) according to internally validated methods. In detail, the characterization of the vitamins included vitamins A, B1, B2, B3, B5, B6, B8, B9, B12, C, and D2. According to De Angelis et al. [28], starch hydrolysis was evaluated using an in vitro enzymatic digestion methodology to compute the hydrolysis index and the related predicted glycemic index [29].

### 2.6. Sensory Characterization of Yogurt-Like

The sensory analyses were carried out on gYL, YL, and a YL fortified with 5% agave syrup and 0.5% of vanilla powder (fYL) by 10 trained panelists (5 men and 5 women; average age: 31 years; range: 24–41 years) with demonstrated abilities and prior expertise in cereal-based and yogurt product assessment. A training session lasting 2 h was performed to choose and describe the attributes to be included in the sessions (color intensity, uniformity in appearance, adherence to spoon, presence of particles, overall odor intensity, pungent odor, sweet taste, salty taste, bitter taste, acidic taste, astringent taste, and earthy taste). All the assessments were conducted in the library of the Department of Soil, Plant, and Food Science at the University of Bari, Italy, as previously described by Elia [30]. The three experimental samples were analyzed in the same assessment and served after 4 h of refrigeration (4 °C) in random order and encoded with three-digit random numbers. All samples were analyzed three times in separate sessions. Each descriptor was evaluated using a 0 to 10 scale, with 10 being the highest perception of the descriptor.

### 2.7. Shelf-Life and Microbial Survival

Aiming at investigating the microbiological quality of the products and the survival of the inoculated strains under refrigerated conditions, YL and fYL were stored at 4 °C for 30 days. The cell densities of presumptive LAB, yeasts, molds, and enterobacteria were enumerated every 5 days during the refrigerated storage period [31]. Moreover, the pH, TTA, and viscosity were also measured, considering these attributes as indicators of the physicochemical properties of the yogurt during storage [32]. All the analyses were performed as previously described (Section 2.3).

### 2.8. Statistical Analysis

All the microbiological, chemical, biochemical, textural, and sensory analyses were carried out in triplicate for each batch of the samples. Data were subjected to Student’s *t*-test between paired groups and one-way ANOVA; pair-comparison of treatment means was achieved by Tukey’s procedure at *p* < 0.05 using the statistical software Statistica 12.5 (StatSoft Inc., Tulsa, OK, USA).

## 3. Results

### 3.1. Optimization of the Yogurt-Like Product

**Figure 1:**
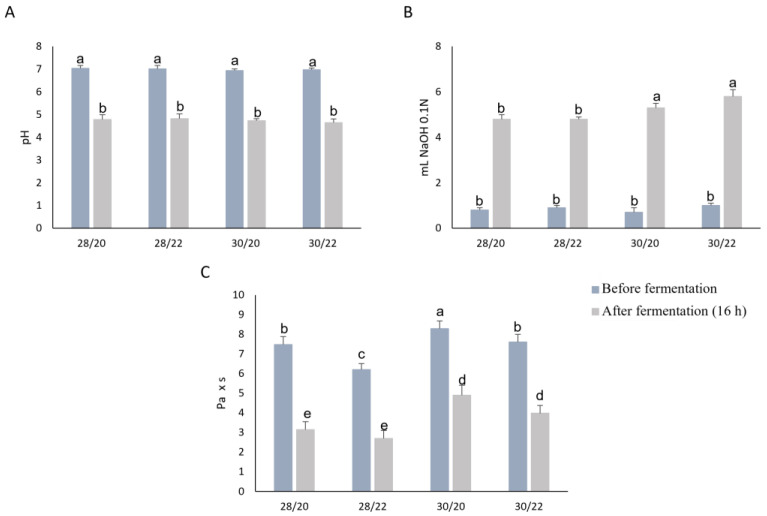
Means (blue and grey bars) and standard deviation (error bars) of the pH (**A**); the total titratable acidity (TTA) (**B**), and viscosity (**C**) of the mixtures (70 g) made from 28 or 30% flour in tap water, in which the hemp flour was added in substitution of 20 or 22% of the rice flour and was subjected to a gelatinization process at 80 °C for 15 min. Analyses were carried out before and after fermentation by *Lactiplantibacillus plantarum* 18S9, *Leuconostoc mesenteroides* 12MM1, and *Leuconostoc pseudomesenteroides* DSM20193 (inoculation density of approximately 6 log cfu/g) at 25 °C for 16 h. ^a–e^ different superscript letters differ significantly (*p* < 0.05).

Viscosity, pH, and TTA were used to define the optimal ratio between the two flours and water and the inclusion of the hemp flour in the yogurt-like product. At the end of the fermentation, the viscosity of the samples containing 20% and 25% flour was too low (below 1 Pa × s); therefore, only 28 and 30% of the total flour contents were considered. Considering that the viscosity of products significantly dropped during fermentation, only the thesis characterized by 28–30% total flour and hemp 20–22% in substitution of the rice flour was further characterized. Figure 1 displays the findings for the three examined parameters (viscosity, pH, and TTA) in samples containing 22.4% rice and 5.6% hemp flour (28/20), 21.84% rice and 6.16% hemp flour (28/22), 24% rice and 6% hemp flour (30/20), and 23.4% rice and 6.6% hemp flour (30/22).

At the end of the gelatinization process, the viscosity of these samples was in the range 6.2 ± 0.3 (28/22)–8.3 ± 0.4 (30/20) Pa × s. No statistically significant difference in terms of pH was found among the gelatinized samples. Similarly, after the fermentation, the pH ranged between 4.65 ± 0.16 and 4.82 ± 0.22, without a statistical difference among the samples, although a modest but significant increase in TTA was found in the samples containing 30% flour with no statistical differences when different fortification levels of hemp flour were used (5.3 ± 0.2 and 5.8 ± 0.4 mL of 0.1 N NaOH). The fermentation process decreased the viscosity mainly when 30% flour in water was used. Indeed, the viscosity of the samples containing 30% total flour (of which 20 and 22% was hemp) decreased by 40% and 48%, respectively. According to the above results, the sample containing 23.4% rice flour and 6.6% hemp flour (30/20) was selected for further analysis.

### 3.2. Kinetics of Growth and Acidification

**Figure 2:**
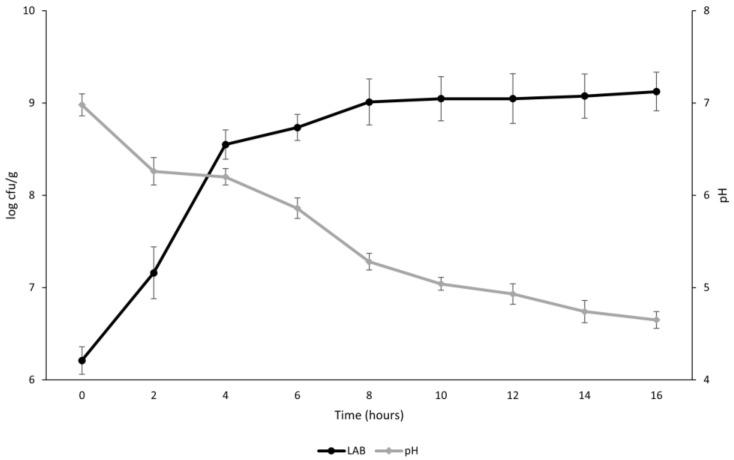
Acidification and lactic acid bacteria growth in gelatinized (80 °C for 15 min) substrates fermented by *Lactiplantibacillus plantarum* 18S9, *Leuconostoc mesenteroides* 12MM1, and *Leuconostoc pseudomesenteroides* DSM20193 (inoculation density of approximately 6 log cfu/g) at 25 °C for 16 h.

In order to confirm the aptitude of the three starters to grow and acidify the gYL, the pH and the LAB cell density were monitored every 2 h during the fermentation (Figure 2). After 4 h of incubation, LAB increased by two logarithmic cycles, while a slightly greater increase was found up until the end of the incubation time (Figure 2).

Therefore, a short lag phase was detected (λ = 0.97 ± 0.21), while high values for μmax and A (0.76 ± 0.10 and 2.41 ± 0.14, respectively) confirmed the aptitude of the mixed starter to grow in the gelatinized formulation. Similarly, acidification kinetics showed results that were consistent with microbial growth, with a latency period value of 0.98 ± 0.18 and Vmax and A values of 0.26 ± 0.04 and 2.42 ± 0.08, respectively. Indeed, the highest decrease in pH was found after 2 h (−0.72), and the decrease during the incubation time was constant (R^2^ = 0.9434).

### 3.3. Microbiological and Biochemical Characterization of the Yogurt-Like Product

Changes in microbial cell density were monitored through the YL-making process. The gelatinization caused a slight decrease in the cell density of all the microbial groups analyzed, with a further change after the fermentation (Table 1). As expected, a significant increase in LAB cell density was found at the end of the 16 h fermentation, reaching a value of 9.10 ± 0.23 log cfu/g. On the contrary, the yeast and enterobacteria cell densities showed a significant decrease (approximately 24 and 52%, respectively), while the molds were not detected after fermentation (Table 1).

YL was characterized by a concentration of lactic acid and acetic acids (12.34 ± 0.33 and 3.78 ± 0.16 mmol/kg, respectively), which were significantly higher than those of gYL, with a fermentation quotient of 3.26 (Table 2). A slight but not significant increase in the peptide’s concentration was also found. gYL and YL showed 7.55 ± 0.34 and 7.96 ± 0.34 mg/g, respectively. Nevertheless, a change in the peptide profile (data not shown) was recorded through reversed-phase fast-performance liquid chromatography (RP-FPLC) analysis. Conversely, the total free amino acids decreased by about 38% at the end of fermentation (Table 2), and, among all the amino acids, the main decreases were found for asparagine and glutamic acid (−110.6 ± 8.7 and −52.6 ± 6.4 mg/kg, respectively), while aspartic acid and ammonia increased (14.3 ± 8.7 and 11.9 ± 8.7 mg/kg, respectively).

The antioxidant activity of WSE and ME were tested against ABTS^+^. No differences between the unfermented and fermented samples were found when ME was used for the assay. Conversely, a slight but significant increase in antioxidant activity was observed in WSE (from 1.49 ± 0.05 of gYL to 1.61 ± 0.08 mmol Trolox eq/g of YL).

### 3.4. Nutritional Characterization of the Yogurt-Like

Fermentation did not result in any significant changes in macronutrient content except for the sugars. Indeed, a significantly higher concentration of glucose was found in YL (0.79 ± 0.08 g/100 g) compared to gYL (0.10 ± 0.07 g/100 g), although this was lower than 1% (*w*/*v*). Overall, the concentration of the total carbohydrates was lower than 17 g/100 g in both gYL and YL (Table 3).

In terms of concentration, protein represented the second macronutrient after carbohydrates, with contents of 3.75 ± 0.31 and 4.19 ± 0.26 in gYL and YL 1.2 g/100 g, respectively. The fats were represented by approximately 80% unsaturated fat. In terms of micronutrients, the fermentation did not change the mineral and vitamin contents of YL. After fermentation, the starch hydrolysis index (and consequently the predicted glycemic index) decreased. In detail, the pGI decreased from 61.17 ± 2.18 to 54.97 ± 3.75 in gYL and YL, respectively.

### 3.5. Monitoring of the Microbial Survival and Main Yogurt-Like Characteristics under Storage Conditions

The technological and microbiological quality of the YL product was monitored during 30 days of storage under refrigerated conditions. All the considered parameters were affected by the storage. Indeed, although the contents remained constant for the first 10 days of storage, the viscosity was subjected to a gradual drop leading to a significant decrease of 37% after 30 days (3.14 ± 0.35 Pa × s). Moreover, the pH decreased, and the TTA increased, reaching values of 3.94 ± 0.18 and 9.4 ± 0.2 mL NaOH 0.1 M after 10 days, and 3.76 ± 0.18 and 12.1 ± 0.3 mL NaOH 0.1 M after 30 days. After 30 days of storage, the cell density of LAB was 8.65 ± 0.35 log cfu/g. After 5 days of storage and throughout the storage period, yeast and enterobacteria were not detected, while the slight growth of molds (1.67 ± 0.58 log cfu/g) was found at the end of the storage period.

### 3.6. Sensorial Evaluation of the Yogurt-Like Product

**Figure 3:**
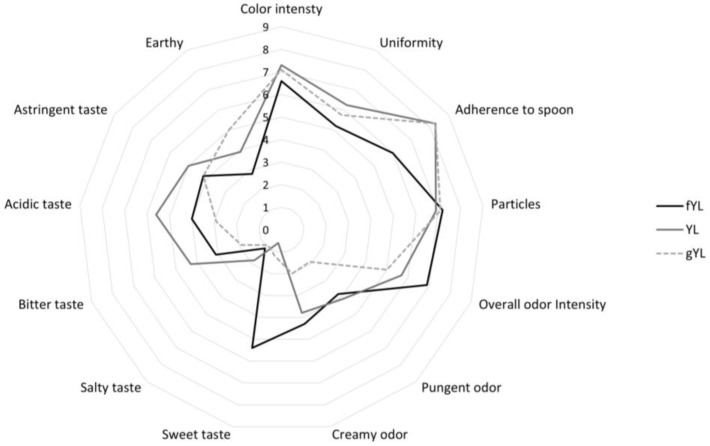
Sensory analysis included gelatinized (80 °C for 15 min) substrates before (gYL) and after fermentation by *Lactiplantibacillus plantarum* 18S9, *Leuconostoc mesenteroides* 12MM1, and *Leuconostoc pseudomesenteroides* DSM20193 (inoculation density of approximately 6 log cfu/g) at 25 °C for 16 h (YL), and the fermented yogurt-like product had 5% agave syrup and 0.5% of vanilla powder (fYL) added.

The sensory analysis of gYL and YL was assessed (Figure 3).

Overall, fermentation caused changes in the sensory profile of the product. Indeed, the flavor and taste attributes were significantly affected. YL was characterized by a pungent odor and a more intense acid taste. Similarly, a creamy odor and sweet taste were much more perceived in YL when compared to gYL. Additionally, the bitter taste perception increased after fermentation. The degree of spoon adhesion was also enhanced through biological acidification, which affected gel formation and maintenance. Indeed, higher values were found for YL when compared to gYL (Figure 3). In order to balance the bitter taste that is typical of hemp flour, the inclusion of 5% agave syrup and 0.5% of vanilla powder in YL (fYL) was considered a valuable option to better meet consumer acceptability. Astringent taste decreased in fYL to the values found in gYL, while bitter and acidic tastes decreased by approximately 30% compared to YL. Moreover, the increases in sweet taste, creamy odor, and overall odor intensity and a further decrease in earthy taste (−22%) were reported in fYL (Figure 3). The color of the three yogurt-like products tested was significantly affected by the inclusion of hemp flour in the formulation, which is dark green. Indeed, all the supplemented products were characterized by green color. Moreover, color intensity was used to describe the variation caused by the fermentation (YL vs. gYL) and addition with natural sweeteners (fYL vs. YL). According to the data, fermentation did not cause significant changes in color intensity, while a slight decrease was found in fYL.

## 4. Discussion

“Healthy living” is the most influential trend identified in the food sector [33] and is concretely interpreted as the increase in consumer demand for less processed foods [34]. Healthy diets include an adequate calorie intake and consist mostly of a variety of plant-based foods, minimal quantities of foods derived from animals, unsaturated fats rather than saturated fats, high fiber content, and low sugar [35]. Therefore, the shifting of consumers into vegetarianism and veganism is continuously growing in popularity [36], and veganism seems to be the largest lifestyle trend of the 21st century [37]. This development provides an opportunity for grain products with additional value to cover the nutritional shortages also confirmed by the industrial success seen in the marketing of plant-based products (including fermented beverages and yogurt), suggesting that innovative applications of cereals, legumes, and pseudocereals may provide an alternative to satisfy consumer expectations [8,13,36]. In addition to a plant-based diet, the “free from” dietary trend is growing in popularity according to the production of “clean label” products [36,38].

In this scenario, hemp represents one of the few innovative food plants which combines positive effects, such as ingredients [39] and environmental sustainability [40]. Both these aspects are associated with the gaining interest in diets rich in plant-based foods and low in animal-derived products [35].

In this work, a YL product, including hemp flour, was designed, and the biotechnological process was set up. Additionally, the main nutritional, functional, and sensorial properties of the prototype were evaluated and compared to an unfermented control. Moreover, the effects on microbiological and structural stability after 30 days under refrigerated storage were investigated.

Preliminarily, the ratio between flour and water, as well as the level of hemp flour fortification, were optimized. Rice flour was previously used to produce PBYL products [41], which was also included in an ice cream formulation [42]. A re-milling of the flour was carried out to obtain a homogeneous mixture after gelatinization. Indeed, smaller granules hydrate better, thus resulting in uniform gelatinization [43]. In general, starchy flours are used at concentrations ranging between 15% and 35% in water suspensions [8]. In this work, a flour percentage range between 20 and 30% in water was evaluated. The use of percentages lower than 25% led to gelatinized substrates with a poor structure which were not suitable for YL production.

Despite the fermentation process carried out by EPS-producing LAB *Le. mesenteroides* 12MM1 and *Le. pseudomesenteroides* DSM20193 [20,23], the viscosity decreased after fermentation (by up to 48%). This effect may be related to the low concentration of sucrose in the formulation, which, being the EPS-production promoter [31,44,45], might be responsible for the low synthesis. Moreover, the synthesis of acetic and lactic acids during fermentation can negatively affect the physicochemical characteristics of gelatinized flour, as was previously found for wheat starches [46].

Nonetheless, the observed viscosity values were greater than those previously found in many plant-based yogurt-like products, including EPS [8,22]. The inclusion of hemp flour in rice-based formulations contributed to viscosity decreases in the gelatinized substrates, both before and after fermentation. Indeed, hemp flour was characterized by low amounts of starch and a high amount of fiber and proteins compared to rice flour. Typically, proteins may affect starch gelatinization, possibly producing a physical matrix that prevents starch from swelling and competing with starch for free water [47,48]. Similarly, the high presence of fibers changes the hydration of starch [47]. No significant differences were found among the inclusion of 20 or 22% hemp flour in the mixtures characterized by 30% total flour in water. Therefore, the formulation with higher amounts of hemp flour was chosen, aiming at a better nutritional value in the final product.

After gelatinization, a significant decrease in the cell density of the endogenous enterobacteria and LAB was found, thus confirming how the gelatinization treatment (in addition to modifying the structure of the starch contained in the flours) allows for a reduction in contaminating micro-organisms [8]. Moreover, the production of organic acids occurring during fermentation led to a pH of approximately 4.5, which represents the value of milk-derived yogurt obtained with *Streptococcus thermophilus* and *Lactobacillus delbrueckii* as starters [49]. The low pH and the very high LAB density (9 log cfu/g) are among the factors that guarantee a long microbial shelf-life for a product. Unexpectedly, a decrease in total free amino acids, mainly due to the marked decrease in the asparagine and glutamic acid contents, and a slight increase in glucose were found after fermentation in YL. Asparagine and glutamine deamination by LAB in dairy products previously demonstrated a decrease in the contents of these amino acids and an increase in aspartate and glutamate [50]. Both amino acids were considered important for decreasing the lag phase of *Lactococcus lactis* [51], while asparagine was found to be essential for the initial growth of *Leuconostoc mesenteroides* [52,53]. Moreover, the increase in ammonia and aspartic acid confirmed the LAB activity related to amino acid catabolism. Similarly, the glucose increment after fermentation could be related to LAB metabolic activity. Indeed, the possibility of hydrolyzing starch was previously demonstrated in LAB, thus defining amylolytic LAB. This peculiar characteristic was found in both *Lactocacillus* and *Leuconostoc* specific strains isolated from different starch-containing sources [54].

In general, the protein concentration of the formulation was not affected by fermentation; however, the nutritional claim “source of protein” could be used given that 17.2% of the energy in YL and 15.6% in gYL is derived from this macronutrient. Moreover, fermentation led to a slight increase in soluble fiber, and both formulations were qualified as “sources of fiber” according to Regulation EC 1924/2006 since they contain more than 1.5 g of fiber per 100 kcal. The micronutrient and vitamin concentrations did not change significantly with fermentation. However, the high concentration of minerals from hemp flour led to a high concentration of magnesium, iron, and zinc in the hemp-based yogurt-like product, which represented respectively 12.5, 9.3, and 8.6% of the reference values for the daily intake reported by the Annex XIII of EU Regulation n. 1169/2011. The pGI of the product was characterized by a low value in gYL and a significant decrease in YL. Usually, because of the resistant-starch conversion from native starch, biological acidification is also linked to a lower starch hydrolysis index and, thus, a lower glycemic index [28]. A pGI of 54.97 ± 3.75 was found in the hemp-containing YL, which was lower than that previously found in YL products made with oat flakes [55], quinoa [56], and emmer [57], while it is comparable with that observed in pulse-derived products [34]. Thanks to its antioxidant properties, hemp flour was previously used to improve the functional features of wheat bread [58]. In this work, and according to previous research [22], a slight but significant increase (+8%) in antioxidant activity toward the ABTS^+^ radical was found in the aqueous extract of the product.

The maintenance of the structure of a PBYL product [10] and the high viability of LAB in yogurt (in general) [59] constitute the main issues during storage. Moreover, the metabolic activity of the starters is related to a further decrease in pH, which can negatively affect the sensorial properties of the product [8]. After 10 days of storage, no changes were detected in the hemp-containing yogurt-like product. Moreover, the cell density of LAB decreased by only 0.5 log cfu/g after 30 days of refrigerated storage, thus confirming the good characteristics of the formulation for being a substrate for the growth and survival of LAB. However, the high survival rate of the starters corresponded to a further decrease in pH during storage, which was lower than 4 after 10 days. The decrease in pH during storage is a well-known phenomenon for yogurt, and it is defined as ‘over acidification’ [60]. Low pH and high acidity could influence the sensorial properties of the product considering the consumer preferences for less acidity and a thicker consistency in commercial yogurt [60]. Among all the sensorial attributes, fermentation led to an increase in both acidic taste and pungent odor. Acidic taste and odor are considered typical for yogurt sensorial profiles, mainly due to the presence of lactic acid, which is positively considered a significant compound that is often claimed to be responsible for giving yogurt a nice flavor [61]. After fermentation, a significant increase in creaminess and a reduction in earthy taste were found. The increase in creaminess perception was positively correlated with high sensorial appreciably and a higher pH in conventional yogurt [62], while, in hemp protein and hemp milk, an earthy taste was previously found to be a negative attribute [63]. However, an increase in bitterness and astringency was detected in YL. Bitterness is identified as a common attribute that is associated with hemp flour [20,64] and, in association with astringency, is usually related to phenolic compounds found in the outer layers of whole grains [65]. Moreover, the concentrates and isolates of plant proteins in water are well known for bitterness and astringency [66]. These two attributes are usually found in gluten-free plant-based products and are overall considered unacceptable by consumers [20,67,68,69]. Therefore, aiming at decreasing these two attributes and increasing the attributes usually related to high consumer acceptability, the inclusion of agave syrup and vanilla powder was tested as a proof-of-concept for the development of a commercial product, thus obtaining a decrease in both bitterness and astringency. Agave syrup is considered a “clean label” ingredient and is made mostly from fructose, thus resulting in a sweeter taste and a lower glycemic index than sucrose [70]. The use of sweeteners, jam, and fruit was previously demonstrated to be effective in improving the sensorial properties of PBYL products [8], and, in the hemp-based yogurt-like product, decreased the earthy, astringent, bitter, and acidic tastes and increased the sweet taste and overall odor intensity.

## 5. Conclusions

In this work, a biotechnological protocol for making a novel PBYL product by using rice and hemp flour and a mixed starter, including three selected LAB, was proposed. The use of hemp flour led to a high fiber (up to *circa* 3%) and protein (up to *circa* 4%) content and a high amount of minerals. Fermentation increased the soluble fiber content and decreased the predicted glycemic index (*circa* 55) when compared to the unprocessed mixture. The high viability of LAB (higher than 8 log cfu/g) and good maintenance of viscosity under refrigerated conditions suggest a predicted shelf-life of 30 days. The sensory properties of the YL product were tested, and the inclusion of agave syrup and vanilla powder was investigated as an option to further improve the product’s palatability. Indeed, fermentation decreased the earthy flavor and increased the acidic and creamy perceptions that are typical of yogurt, while the supplementation with sweeteners decreased the off flavors (e.g., astringent and bitter) usually associated with PBYL products and increased the perception of sweetness.

This work demonstrated that hemp flour is an ingredient that can improve the nutritional balance of other plant-based foods (e.g., cereal-based) and that fermentation with selected LAB represents a sustainable and effective option to exploit its potential.

## Figures and Tables

**Table 1:** Microbiological characterization of the yogurt-like product made with hemp and rice flours after the preparation of the liquid mixture (ngYL) and after gelatinization for 15 min at 80 °C (gYL), as well as at the end of fermentation for 16 h at 25 °C by *Lactiplantibacillus plantarum* 18S9, *Leuconostoc mesenteroides* 12MM1, and *Leuconostoc pseudomesenteroides* DSM20193 (YL).

	LAB (log cfu/g)	Yeasts (log cfu/g)	Molds (log cfu/g)	Enterobacteria (log cfu/g)
ngYL	3.98 ± 0.27 ^c^	2.75 ± 0.20 ^a^	2.37 ± 0.47 ^a^	2.78 ± 0.17 ^a^
gYL	3.21 ± 0.38 ^b^	2.63 ± 0.15 ^a^	2.11 ± 0.34 ^a^	2.30 ± 0.21 ^b^
YL	9.10 ± 0.23 ^a^	2.09 ± 0.21 ^b^	n.d.	1.32 ± 0.18 ^c^

The data are the means of three independent experiments ± standard deviations (*n* = 3). ^a–c^ Values in the same row with different superscript letters differ significantly (*p* < 0.05).

**Table 2:** Biochemical characterization of the yogurt-like product made with hemp and rice flours after gelatinization for 15 min at 80 °C (gYL) and at the end of the fermentation for 16 h at 25 °C by *Lactiplantibacillus plantarum* 18S9, *Leuconostoc mesenteroides* 12MM1, and *Leuconostoc pseudomesenteroides* DSM20193 (YL).

	gYL	YL
Lactic acid (mmol/kg)	0.25 ± 0.04 ^b^	12.34 ± 0.33 ^a^
Acetic acid (mmol/kg)	0.14 ± 0.05 ^b^	3.78 ± 0.16 ^a^
TFAA (mg/kg)	726 ± 28 ^a^	453 ± 15 ^b^
Radical scavenging activity ME (mmol Trolox eq/g)	3.72 ± 0.18 ^a^	3.76 ± 0.19 ^a^
Radical scavenging activity WSE (mmol Trolox eq/g)	1.49 ± 0.05 ^b^	1.61 ± 0.08 ^a^

The data are the means of three independent experiments ± standard deviations (*n* = 3). ^a–b^ Values in the same row with different superscript letters differ significantly (*p* < 0.05).

**Table 3:** Nutritional characterization of the yogurt-like product made with hemp and rice flours after gelatinization for 15 min at 80 °C (gYL) and at the end of the fermentation for 16 h at 25 °C by *Lactiplantibacillus plantarum* 18S9, *Leuconostoc mesenteroides* 12MM1, and *Leuconostoc pseudomesenteroides* DSM20193 (YL).

	gYL	YL
Ash (% d.m.)	2.55 ± 0.06 ^a^	2.54 ± 0.06 ^a^
Umidity (%)	75.4 ± 5.7 ^a^	75.1 ± 5.6 ^a^
Total carbohydrates (g/100 g)	16.8 ± 0.9 ^a^	16.5 ± 1.0 ^a^
Fructose (g/100 g)	n.d.	0.01 ± 0.01
Glucose (g/100 g)	0.10 ± 0.07 ^b^	0.79 ± 0.08 ^a^
Maltose (g/100 g)	n.d.	n.d.
Sucrose (g/100 g)	n.d.	n.d.
Sugar (g/100 g)	0.10 ± 0.07 ^b^	0.80 ± 0.08 ^a^
Total fat (g/100 g)	1.0 ± 0.3 ^a^	1.2 ± 0.2 ^a^
Saturated fat (g/100 g)	0.18 ± 0.04 ^a^	0.20 ± 0.03 ^a^
Nitrogen (% N t.q.)	0.60 ± 0.05 ^a^	0.67 ± 0.04 ^a^
Proteins (% N*6.25 t.q.)	3.75 ± 0.31 ^a^	4.19 ± 0.26 ^a^
Energy (kJ/100 g)	408 ± 18 ^a^	413 ± 21 ^a^
Fiber (g/100 g)	2.6 ± 0.5 ^a^	2.7 ± 0.5 ^a^
Insoluble fiber (g/100 g)	2.5 ± 0.3 ^b^	2.1 ± 0.4 ^b^
Soluble fiber (g/100 g)	0.2 ± 0.2 ^b^	0.5 ± 0.1 ^a^
Salt (g/100 g)	0.001 ± 0.001 ^a^	0.001 ± 0.001 ^a^
Calcium (mg/kg)	516 ± 47 ^a^	468 ± 32 ^a^
Iron (mg/kg)	13.1 ± 1.3 ^b^	16.3 ± 1.2 ^a^
Phosphorus (mg/100 g)	107 ± 9 ^a^	100 ± 11 ^a^
Magnesium (mg/kg)	475 ± 25 ^a^	468 ± 19 ^a^
Potassium (mg/kg)	1155 ± 38 ^a^	1208 ± 42 ^a^
Zinc (mg/kg)	8.60 ± 0.24 ^a^	7.71 ± 0.13 ^a^
Total tocopherols (mg/kg)	<0.5	<0.5
Vit A (ug/100 g)	<10	<10
Vit. B1 (ug/kg)	0.102 ± 0.023 ^a^	0.075 ± 0.031 ^a^
Vit. B2 (ug/kg)	0.031 ± 0.010 ^a^	0.030 ± 0.010 ^a^
Vit. B3 (ug/kg)	0.595 ± 0.023 ^a^	0.475 ± 0.034 ^b^
Vit. B5 (ug/kg)	1438 ± 42 ^a^	1521 ± 39 ^a^
Vit. B6 (ug/kg)	0.106 ± 0.021 ^a^	0.101 ± 0.009 ^a^
Vit B8 (ug/kg)	<10	<10
Vit. B9 (ug/kg)	<20	<20
Vit. B12 (ug/kg)	<5	<5
Vit. C (mg/100 g)	<1	<1
Vit. D2 (mg/kg)	<0.002	<0.002

The data are the means of three independent experiments ± standard deviations (*n* = 3). ^a–b^ Values in the same row with different superscript letters differ significantly (*p* < 0.05).

## Data Availability

Data is contained within the article or Supplementary Material.

## Supplementary Materials

The following supporting information can be downloaded at: https://www.mdpi.com/article/10.3390/foods12030485/s1, Table S1: Flour (rice and hemp) mixtures used in the production of mixtures for the preliminary evaluation of viscosity after gelatinization (80 °C for 15 min).
