# Supplementary material for: Design of a Plant-Based Yogurt-Like Product Fortified with Hemp Flour: Formulation and Characterization
Source: Foods. 2023 Jan 20;12(3):485. doi: 10.3390/foods12030485 (PMC9914809; doi:10.3390/foods12030485)
Supplement: Supplementary file 1 [file foods-12-00485-s001.zip › foods-2173717-supplementary .pdf]

Supplementary Table S1. Flour (rice and hemp) mixtures used in the production of mixtures for the preliminary evaluation of viscosity after gelatinization (80 °C for 15 min).

	Total amount of flour			
Hemp flour in substitution	20%	25%	28%	30%
15%	Rice: 17g/100g<br>Hemp: 3g/100g<br>Water: 80g/100g	Rice: 21.25g/100g<br>Hemp: 3.75g/100g<br>Water: 75g/100g	Rice: 23.8g/100g<br>Hemp: 4.2g/100g<br>Water: 72g/100g	Rice: 25.5g/100g<br>Hemp: 4.5g/100g<br>Water: 70g/100g
20%	Rice: 16g/100g<br>Hemp: 4g/100g<br>Water: 80g/100g	Rice: 20g/100g<br>Hemp: 5g/100g<br>Water: 75g/100g	Rice: 22.4g/100g<br>Hemp: 5.6g/100g<br>Water: 72g/100g	Rice: 24g/100g<br>Hemp: 6g/100g<br>Water: 70g/100g
22%	Rice: 15.6g/100g<br>Hemp: 4.4g/100g<br>Water: 80g/100g	Rice: 19.5g/100g<br>Hemp: 5.5g/100g<br>Water: 75g/100g	Rice: 21.84g/100g<br>Hemp: 6.16g/100g<br>Water: 72g/100g	Rice: 23.4g/100g<br>Hemp: 6.6g/100g<br>Water: 70g/100g
25%	Rice: 15g/100g<br>Hemp: 5g/100g<br>Water: 80g/100g	Rice: 18.75g/100g<br>Hemp: 6.25g/100g<br>Water: 75g/100g	Rice: 21g/100g<br>Hemp: 7g/100g<br>Water: 72g/100g	Rice: 22.5g/100g<br>Hemp: 7.5g/100g<br>Water: 70g/100g
